# Complete Genome Sequence of Ferriphaselus amnicola Strain OYT1, a Neutrophilic, Stalk-Forming, Iron-Oxidizing Bacterium

**DOI:** 10.1128/MRA.00911-18

**Published:** 2018-09-27

**Authors:** Shingo Kato, Masahiro Yuki, Takashi Itoh, Moriya Ohkuma

**Affiliations:** a Japan Collection of Microorganisms, RIKEN BioResource Research Center, Tsukuba, Ibaraki, Japan; Iowa State University

## Abstract

Ferriphaselus amnicola is a freshwater, neutrophilic, iron-oxidizing bacterium that produces extracellular twisted-ribbon-like iron biominerals called stalks. Here, we report the 2.72-Mb closed genome sequence of F. amnicola strain OYT1, which was isolated from iron oxide deposits at a groundwater stream in Japan.

## ANNOUNCEMENT

In the 1830s, a freshwater, stalk-forming, iron-oxidizing bacterium (FeOB), Gallionella ferruginea, was described (1), and it is one of the oldest records of a bacterial species. Several physiological and morphological characterizations of G. ferruginea have been reported since then (2, 3); however, G. ferruginea is not currently available in any culture collection. Previously, we isolated another stalk-forming FeOB strain, OYT1^T^ (JCM 18545^T^=DSM 26810^T^), from a groundwater seep in Japan (4) and proposed the name Ferriphaselus amnicola gen. nov., sp. nov. (5). F. amnicola belongs to the class Betaproteobacteria and shows a 92.9% 16S rRNA gene sequence similar to that of G. ferruginea. The draft genome sequence (2.68 Mb, 23 contigs) of the strain was already determined by 454 pyrosequencing and Ion PGM sequencing, which indicated the presence of putative genes involved in carbon fixation, iron oxidation, and stalk formation (6). However, its complete genome has not been reported so far.

Here, we present the complete genome sequence of F. amnicola strain OYT1. The cultivation of the strain and the DNA extraction from the culture have been reported previously (6). In brief, F. amnicola strain OYT1 was cultured in a mineral medium with agarose-stabilized FeS as the Fe(II) source at pH 6.2 and 25°C. Using the same DNA extract, we resequenced its genome on a MiSeq platform (Illumina, USA) and on a MinION device (Oxford Nanopore Technologies [ONT], United Kingdom) with an R9.4 flow cell (ONT). Sequencing libraries were constructed using a QIAseq FX DNA library kit (Qiagen, Germany) for the Illumina sequencing and a rapid sequencing kit (SQK-RAD004, ONT) for the ONT sequencing. We obtained 9,621,832 Illumina paired-end short reads (mean length, 223 bp) with a total of 4,295,964,156 bp and 610,696 ONT long reads (mean length, 1,717 bp) with a total of 1,048,892,817 bp. *De novo* assembly of the genome using both the short and long reads was performed using Unicycler version 0.3.0b (7), resulting in a circular contig of 2,717,229 bp with a G+C content of 55.9%. Genome annotation was performed using Prokka version 1.12 (8) and the Rapid Annotations using Subsystems Technology (RAST) server version 2.0 (9), and the annotated genome was manually curated.

**FIG 1:**
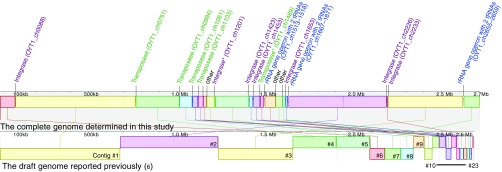
Comparison between the complete genome determined in this study and the previously reported draft genome of F. amnicola strain OYT1 (6). Three copies of the rRNA gene operon with 2 tRNAs and over 10 CDSs for transposase and integrase were detected in the genome. Colored regions of the complete genome represent the homologous regions to the previously reported contigs. Asterisks (*) indicate partial CDSs.

The genome contained 2,683 protein-coding regions (CDSs), 3 copies of rRNA gene operons (5S, 16S, and 23S), and 50 coding regions of tRNAs. In addition to the rRNA genes, 4 copies of CDSs for transposase (identical to each other, 1,026 bp) and 7 copies of CDSs for integrase (identical to each other, 1,515 bp) were encoded in the genome. These CDSs probably caused the previous failure of the finished genome assembly, which used only short reads (<400 bp) produced by 454 pyrosequencing and Ion PGM sequencing (6), as supported by the mapping of the previously reported contigs on the complete genome (Fig. 1) using Mauve version 2015-02-25 (10). The presence of these CDSs related to mobile genetic elements suggests that transposons and integrons have played a role in the evolution of the F. amnicola genome.

### Data availability.

The complete genome sequence determined in the present study has been deposited in DDBJ/ENA/GenBank under the accession no. AP018738, which is linked to the BioProject accession no. PRJDB3480.

## References

[1] Ehrenberg CG 1838 Die infusionsthierchen als vollkommene organismen: ein blick in das tiefere organische leben der natur. Verlag von Leopold Voss, Leipzig.

[2] Vatter AE, Wolfe RS 1956 Electron microscopy of *Gallionella ferruginea*. J Bacteriol 72:248–252. 13366907 10.1128/jb.72.2.248-252.1956 PMC357887

[3] Hallbeck L, Ståhl F, Pedersen K 1993 Phylogeny and phenotypic characterization of the stalk-forming and iron-oxidizing bacterium *Gallionella ferruginea*. J Gen Microbiol 139:1531–1535. doi:10.1099/00221287-139-7-1531. 8371116

[4] Kato S, Chan C, Itoh T, Ohkuma M 2013 Functional gene analysis of freshwater iron-rich flocs at circumneutral pH and isolation of a stalk-forming microaerophilic iron-oxidizing bacterium. Appl Environ Microbiol 79:5283–5290. doi:10.1128/AEM.03840-12. 23811518 PMC3753938

[5] Kato S, Krepski S, Chan C, Itoh T, Ohkuma M 2014 *Ferriphaselus amnicola* gen. nov., sp. nov., a neutrophilic, stalk-forming, iron-oxidizing bacterium isolated from an iron-rich groundwater seep. Int J Syst Evol Microbiol 64:921–925. doi:10.1099/ijs.0.058487-0. 24425821

[6] Kato S, Ohkuma M, Powell DH, Krepski ST, Oshima K, Hattori M, Shapiro N, Woyke T, Chan CS 2015 Comparative genomic insights into ecophysiology of neutrophilic, microaerophilic iron oxidizing bacteria. Front Microbiol 6:1265. doi:10.3389/fmicb.2015.01265. 26617599 PMC4643136

[7] Wick RR, Judd LM, Gorrie CL, Holt KE 2017 Unicycler: resolving bacterial genome assemblies from short and long sequencing reads. PLoS Comput Biol 13:e1005595. doi:10.1371/journal.pcbi.1005595. 28594827 PMC5481147

[8] Seemann T 2014 Prokka: rapid prokaryotic genome annotation. Bioinformatics 30:2068–2069. doi:10.1093/bioinformatics/btu153. 24642063

[9] Aziz R, Bartels D, Best A, DeJongh M, Disz T, Edwards R, Formsma K, Gerdes S, Glass E, Kubal M, Meyer F, Olsen G, Olson R, Osterman A, Overbeek R, McNeil L, Paarmann D, Paczian T, Parrello B, Pusch G, Reich C, Stevens R, Vassieva O, Vonstein V, Wilke A, Zagnitko O 2008 The RAST server: Rapid Annotations using Subsystems Technology. BMC Genomics 9:75. doi:10.1186/1471-2164-9-75. 18261238 PMC2265698

[10] Darling AE, Mau B, Perna NT 2010 progressiveMauve: multiple genome alignment with gene gain, loss and rearrangement. PLoS One 5:e11147. doi:10.1371/journal.pone.0011147. 20593022 PMC2892488

